# Supplementary material for: Mechanical Regulation of Oral Epithelial Barrier Function
Source: Bioengineering (Basel). 2023 Apr 25;10(5):517. doi: 10.3390/bioengineering10050517 (PMC10215350; doi:10.3390/bioengineering10050517)
Supplement: Supplementary file 1 [file bioengineering-10-00517-s001.zip › Supplementary Materials.pdf]

Supplementary Materials

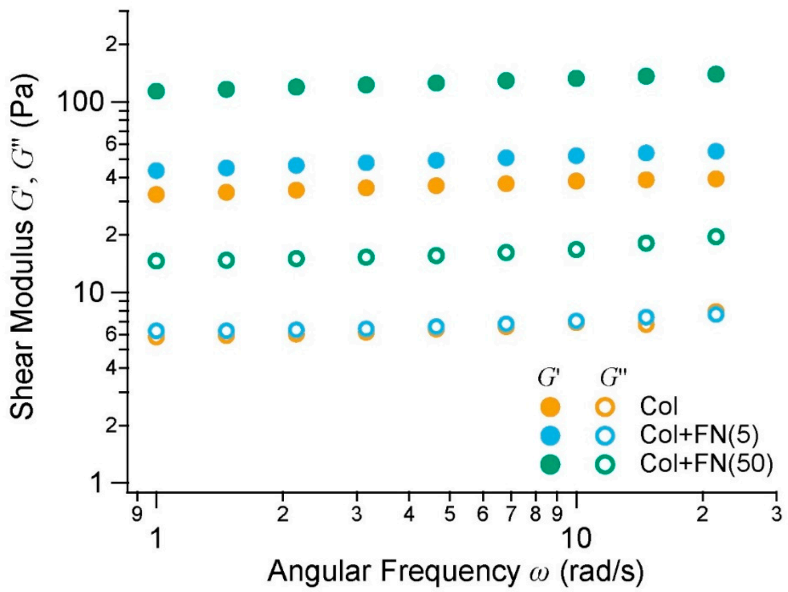

Figure S1. Graph of shear modulus for Col, Col+FN(5) and Col+FN(50) conditions at different frequencies.

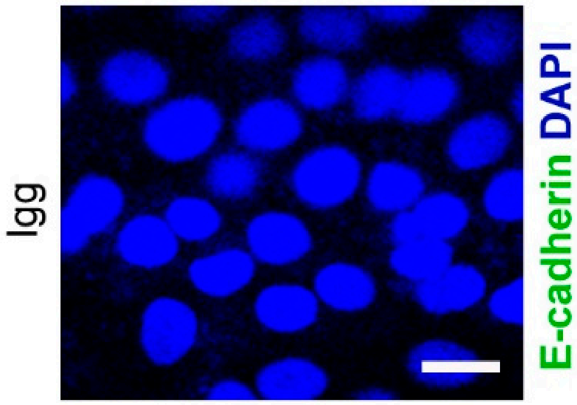

Figure S2. Negative control for staining IgG in HIGKs (Scale bar, 10 μm).

Table S1. PCR primers used in this study.

Gene name	Sequences
human CDH1	Forward 5'-GCC TCC TGA AAA GAG AGT GGA AG-3'
	Reverse 5'-TGG CAG TGT CTC TCC AAA TCC G-3'
human ZO1	Forward 5'-GTC CAG AAT CTC GGA AAA GTG CC-3'
	Reverse 5'-CTT TCA GCG CAC CAT ACC AAC C-3'
human Occludin	Forward 5'-ATG GCA AAG TGA ATG ACA AGC GG-3'
	Reverse 5'-CTG TAA CGA GGC TGC CTG AAG T-3'
human ITGB1	Forward 5'-GGA TTC TCC AGA AGG TGG TTT CG-3'
	Reverse 5'-TGC CAC CAA GTT TCC CAT CTC C-3'
human ITGA6	Forward 5'-CGA AAC CAA GGT TCT GAG CCC A-3'
	Reverse 5'-CTT GGA TCT CCA CTG AGG CAG T-3'
human Lamin 5	Forward 5'-GTC ACA GAG CAG GAG GTG GCT-3'
	Reverse 5'-GCT TCT GTC AAG ACT CTC CAG G-3'
human 18S	Forward 5'-CGG CTA CCA CAT CCA AGG AA-3'
	Reverse 5'-GCT GGA ATT ACC GCG GCT-3'

Abbreviation: CDH1, epithelial cadherin (E-cadherin); ZO1, Zonula occludens-1 (Tight junction protein-1); ITGB1, Integrin beta-1; ITGA6, Integrin alpha-6, 18S, 18S ribosomal RNA

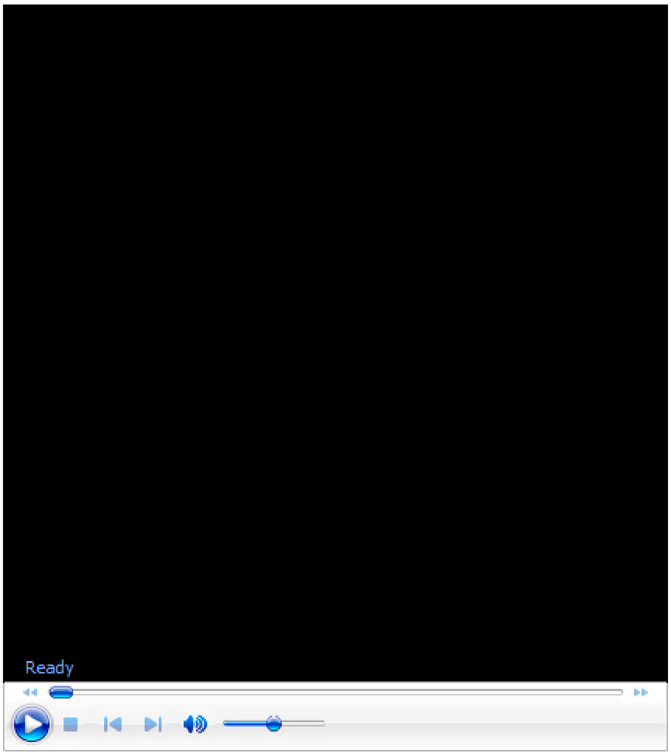

Video S1. Representative video for 70 kDa dextran permeability assay for 3 mg/mL collagen gel in the presence of mechanical stress (0.1 kPa).
